# What the Radiologist Needs to Know About Sport Hernias: A Systematic Review of the Current Literature

**DOI:** 10.3390/diagnostics15060785

**Published:** 2025-03-20

**Authors:** Gian Nicola Bisciotti, Andrea Bisciotti, Alessandro Bisciotti, Alessio Auci

**Affiliations:** 1 Kinemove Rehabilitation Centers, 54027 Pontremoli, Italy; 2 Orthopaedics of the Knee and Sport Traumatology Unit, IRCSS Humanitas Research Hospital, 20089 Rozzano, Italy; andrea.bisciotti@humanitas.it (A.B.); alessandro.bisciotti@icloud.com (A.B.); 3 Azienda USL Toscana Nord-Ovest, 54100 Marina di Massa, Italy; alessioauci@gmail.com

**Keywords:** sport hernia, occult hernia, incipient hernia, weakness of the inguinal canal posterior wall, groin pain syndrome

## Abstract

**Introduction:** The sports hernia (SH) is one of the most important causes of groin pain syndrome (GPS). However, despite its importance in GPS etiopathogenesis, SH is one of the least understood and poorly defined clinical conditions in sports medicine. The aim of this systematic review is to clearly define SH from a radiological point of view and to clarify the relationship between the radiological presentation of SH and its clinical manifestation. **Methods:** The PubMed/MEDLINE, Scopus, ISI, Cochrane Database of Systematic Reviews, and PEDro databases were consulted for systematic reviews on the role of SH in the onset of GPS. The inclusion and exclusion criteria were based on PICO tool. **Results:** After screening 560 articles, 81 studies were included and summarized in this systematic review. All studies were checked to identify any potential conflict of interest. The quality assessment of each individual study considered was performed in agreement with the Joanna Briggs Institute quantitative critical appraisal tools. **Conclusions:** The correct definition of SH is “weakness of the posterior wall of the inguinal canal”, which, in response to a Valsalva maneuver, forms a bulging that compresses the nerves passing along the inguinal canal. Thus, from an anatomical point of view, SH represents a direct inguinal hernia “in fieri”. Furthermore, an excessive dilation of the external inguinal ring represents an indirect sign of possible posterior inguinal canal wall weakness.

## 1. Introduction

The so-called sports hernia (SH) was first described by Nesovic in 1987 [1], who also described the first surgical technique specifically dedicated to this clinical situation [2,3]. The original surgical procedure, adopted by Nesovic, involved an abdominal approach transferring the lateral rectus abdominus muscle towards the inguinal ligament thus achieving a reduction in Hasselbach’s triangle and a reinforcement of the posterior inguinal wall [2,3]. It has been for more than 30 years that a pathological condition of the posterior wall of the inguinal canal has been recognized as one of the major causes of the onset of groin pain syndrome (GPS) [4], yet, despite this, SH paradoxically represents one of the least understood and poorly defined clinical conditions in sport medicine [5]. In fact, the definitions of SH range from the description of a chronic GPS to that of a groin disruption due to pelvic functional instability [6,7,8,9]. Even today, the term “SH” is considered a synonym of athletic pubalgia and, therefore, another term used by some authors to describe GPS [9,10]. In particular, the term SH has been confused with that of athletic pubalgia because originally the term SH meant a posterior inguinal wall weakness due a disruption of transversalis fascia or external and internal oblique and transverse muscle deficiency [11,12,13]. Subsequently, since the anatomical location of the damage was the subject of strong discussion, the various authors began to use the term SH in a generic way to generally mean pain in the groin area [7].

Thus, in light of this terminological confusion, it should be remembered for precision’s sake that “SH” is not an interchangeable term for GPS but corresponds to a very specific anatomical condition [5,14,15,16].

From an epidemiological point of view, SH is predominant in males, with the male population accounting for approximately 90% of all cases [5,17]. This higher incidence in males is justified by the fact that several anatomical characteristics of the female pelvis and inguinal canal act as natural anatomical factors that prevent SH onset. However, some studies conducted on sports populations of both sexes show that the incidence of SH in the sports population is substantially identical in males and females [18,19,20]. This apparent contradiction can be explained, at least partially, by the fact that exploration of the superficial inguinal ring is more difficult in females than in men and this compromises SH detection [21].

As far as sports activities are concerned, SH is mostly found in sports that involve quick changes in direction, sprinting, kicking moves, and violent torsional movements of the pelvis such as football, hockey, and rugby [22]. It is important to underline the fact that, although inguinal hernia and SH represent two different clinical situations, their biological etiopathogenesis is, on the contrary, similar. Indeed, several studies demonstrate that an augmentation of the levels of matrix metalloproteinases (MMPs)-1-2-9-13 and a reduction in the levels of the tissue inhibitor metalloproteinases (TIMPs)-1-2-3 may play a role in the formation of both inguinal hernias and SH [22,23,24,25]. In fact, a loss of structural integrity of the extracellular matrix in an area subjected to mechanical stress, such as the transversalis fascia, would cause a decrease in its resilience and favor the onset of both SH and inguinal hernia [22,23,24,25]. Therefore, both inguinal hernias and SH are not to be considered solely local anatomical damage but rather the consequence of a systemic disease [22,23,24,25]. Several authors have recently considered the possibility of a genetic predisposition for developing inguinal hernias and have focused their attention on several specific loci in the regions of EFEMP1, WT1, EBF2, ADAMTS6, ZC3H11B, and MHC [22,25,26]. In the light of these results, it is legitimate to ask whether there could also be a genetic predisposition for SH. If so, this could represent a further perspective on the tendency for developing groin pathologies and, consequently, GPS. This is a particularly interesting aspect especially for the field of Sports Medicine.

From a clinical point of view, SH can be defined as an activity-related pathology [4,10,27]. Indeed, patients typically experience pain during, or immediately after, physical activity and find relief only upon resting [4,10,27]. The pain felt is localized in the groin area but can radiate to the scrotum and can be exacerbated, not only by sports activities but by sit-ups, coughing, sneezing and by all the movements that cause an increase in intra-abdominal pressure [10,28]. SH is bilateral in approximately 14% of cases in the male sports population [19] and in approximately 27% of cases in the female sports population [20].

During physical examination, diagnosis of SH may be formulated if at least three of the following five points are present [29]:

(1) Pinpoint tenderness during the palpation of the pubic tubercle at the conjoint tendon insertion.
(2) Tenderness over the deep inguinal ring.
(3) Pain and dilation of the external ring during the inspection maneuver in absence of a palpable hernia.
(4) Pain during the palpation of the origin of the adductor longus tendon.
(5) Dull, diffuse pain often radiating to the perineum, scrotum, and the inner thigh.

However, since the fundamental characteristic of SH is the absence of a palpable hernia during clinical examination of the inguinal canal, its diagnosis is essentially based on imaging examination and specifically on dynamic ultrasonography examination (USD) [5,16,30].

Although the etiological causes of GPS are extremely numerous and can be divided into substantially 12 nosological categories for a total of 67 different clinical situations [5], the main and most frequent differential diagnoses for a suspected case of SH are as follows:

(i) Inguinal hernia [31].
(ii) Femoral hernia [32].
(iii) Adductors tendinopathy [33].
(iv) Pre-pubic aponeurotic complex injuries [5,20,34].
(v) Adductor injuries [35].
(vi) Pubic osteopathy [36].
(vii) Iliopsoas tendinopathy [37].

As for treating SH, surgery is the best recommendation [5], especially for sports patients, since conservative treatment of SH has yielded unsatisfactory outcomes [8,38]. The surgical techniques used for the reinforcement of the posterior wall of the inguinal canal are divided into two categories: open techniques, with or without the use of mesh and with or without neurectomy [39,40], and laparoscopic techniques, which always involve the use of mesh [41]. The two types of techniques have similar outcomes [42]. The return to play period is approximately 12 weeks [43] and the percentage of patients who return to their pre-injury level of sporting activity is between 80 and 95% [44,45,46].

In the current literature, one of the most important, critical, and obscure points is the lack of consensus concerning the radiological definition of SH [5,16]. This lack of a clear radiological, and consequently, clinical definition, complicates the situation further, making diagnosis and formulation of a correct treatment difficult for the clinician [47]. The aim of this systematic review is to objectively define SH from a radiological point of view as well as to discuss the relationship between the radiological and clinical manifestation of SH.

## 2. Material and Methods

### 2.1. Aim of the Current Systematic Review

This systematic review was conducted in accordance with the PRISMA (Preferred Reporting Items for Systematic Reviews and MetaAnalysis) guidelines [48]. The protocol of this study is registered with the PROSPERO register for systematic reviews (Number CRD 42024623094).

### 2.2. Data Extraction and Quality Assessment

The PubMed/MEDLINE, Scopus, ISI, Cochrane Database of Systematic Reviews, and PEDro databases were consulted for systematic reviews on the role of SH in the onset of GPS, in order to guarantee the originality of this systematic review. After this initial verification, three authors (GNB, AB, and AA) independently screened the literature using a string of keywords: “sport hernia”, “inguinal posterior wall weakness”, “ultrasound”, “dynamic ultrasound”, “magnetic resonance imaging”, “pelvic anatomy”, “inguinal anatomy”, fittingly connected by Boolean operators. When appropriate, medical subject headings (MeSH) and wild-card options were used.

**Figure 1:**
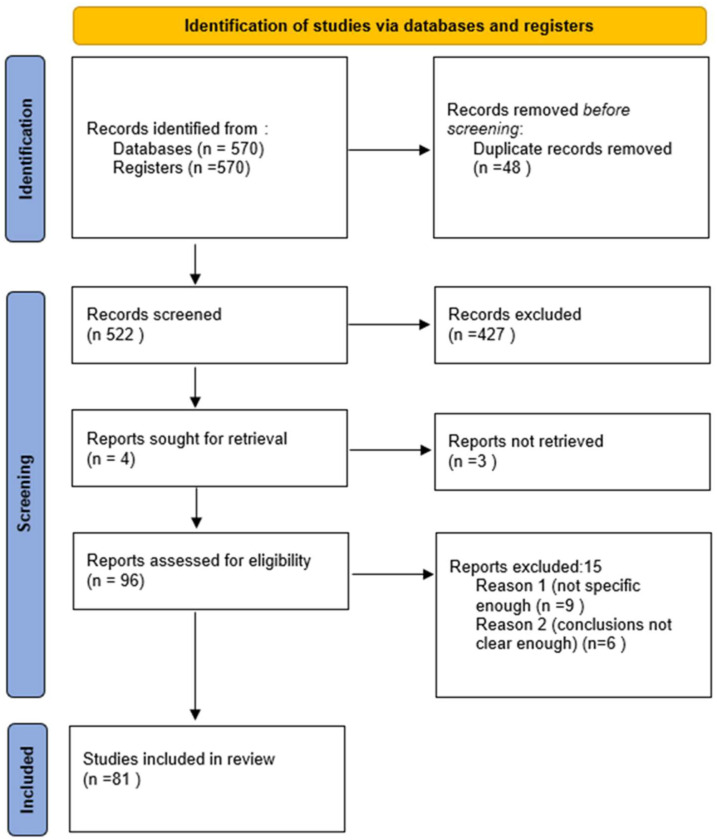
The PRISMA flow diagram of the study search and selection procedure.

Furthermore, target journals were reviewed, in order to collate the maximum number of relevant articles. This phase of research spanned the period 26 October 2024–15 November 2024. Neither data restriction nor language limitation were applied. “Grey literature”, i.e., conference accounts, abstracts, thesis, and unpublished reports, was not taken into account. Cross references from the selected articles were screened to verify their possible relevance. All double citations were removed. For each article, the relevant information was extracted and recorded on an ad hoc Excel spreadsheet. The PRISMA flow diagram of the study search and selection procedure is shown in Figure 1. The quality assessment of each individual study considered was performed in agreement with the Joanna Briggs Institute (JBI) quantitative critical appraisal tools [49] and the results are shown in Table 1.

### 2.3. Search Strategy Items Details

Databases consulted: PubMed/MEDLINE, Scopus, ISI, Cochrane Database of Systematic Reviews, and PEDro.

Search string: (sport hernia OR inguinal posterior wall weakness) AND (ultrasound OR dynamic ultrasound OR magnetic resonance imaging) AND (pelvic anatomy OR inguinal anatomy). The inclusion and exclusion criteria were based on PICO tool [50].

### 2.4. Inclusion Criteria

P: randomized controlled trials, case series studies, cross-sectional studies, cohort studies, systematic review, narrative review, prospective studies, retrospective studies, comparative studies, multicenter studies, observational cross-sectional studies, and case reports focused on imaging sport hernias or inguinal posterior wall weakness.

I: anatomical and clinical studies focused on pelvic anatomy and inguinal anatomy taking into account sport hernias or inguinal posterior wall weakness.

C: comparison between the different imaging methods for detecting sport hernias or inguinal posterior wall weakness.

O: outcome in terms of diagnostic validity of the imaging method.

### 2.5. Exclusion Criteria

P: randomized controlled trials, case series studies, cross-sectional studies, cohort studies, systematic review, narrative review, prospective studies, retrospective studies, comparative studies, multicenter studies, observational cross-sectional studies, and case reports focused on sport hernia- or inguinal posterior wall weakness onset without considering imaging methods.

I: anatomical and clinical studies that did not take into account sport hernias or inguinal posterior wall weakness.

C: studies in which the imaging methods for sport hernias or inguinal posterior wall weakness detection are missing.

O: lack of outcome concerning the diagnostic validity of the imaging method.

### 2.6. Statistical Analysis

Since this systematic review is purely descriptive in nature, no quantitative statistical analysis was performed.

### 2.7. Results of Systematic Review

After screening 560 articles, 81 studies were included and summarized in this systematic review (Figure 1). All studies were checked to identify any potential conflicts of interest. The study design, level of evidence, JBI score, risk of bias, and synthesis of the study concerning each considered study are shown in Table 1.

### 2.8. Study Design

Among the studies selected, there were the following:

18 systematic reviews [5,6,8,9,10,16,19,23,25,28,29,42,46,51,52,53,54,55];

22 narrative reviews [1,4,17,30,31,34,35,36,37,40,43,47,56,57,58,59,60,61,62,63,64,65];

19 case series [2,3,11,14,15,18,20,26,33,66,67,68,69,70,71,72,73,74,75];

5 prospective studies [21,24,38,41,76];

6 retrospective case series [27,32,39,44,77,78];

4 clinical reviews [7,12,13,22];

2 observational cross-sectional studies [79,80];

3 retrospective case–control study [45,81,82];

1 case–control study [83];

1 case report [84].

**Table 1:** Study design, level of evidence, JBI score, risk of bias, and synthesis of the study concerning each considered study. Concerning the risk of bias, if the studies satisfied ≥75% of the requested criteria, the risk of bias was considered low; if the criteria were satisfied by a percentage of 60–74%, the risk of bias was considered moderate; finally, if the criteria were satisfied for <60%, the risk of bias was considered high [49].

Reference	Study Design	Level of Evidence	JBI Score	Risk of Bias	Synthesis of the Study
Bisciotti et al., 2023 [5]	Systematic review	I	90/100	Low	Groin Pain Syndrome Italian Consensus update 2023
Paajanen et al., 2015 [6]	Systematic review	I	78/100	Low	Laparoscopic treatment of long-standing groin pain in athletic population
Jørgensen et al., 2019 [8]	Systematic review	I	66/100	Moderate	Treatment of longstanding groin pain syndrome
Kraeutler et al., 2021 [9]	Systematic review	I	81/100	Low	The difference in Terminology, Surgical Techniques, Preoperative Diagnostic Measures, and Geographic Differences in the Treatment of Athletic Pubalgia
Kopscik et al., 2023 [10]	Systematic review	I	90/100	Low	Sport hernia clinical approach
Bisciotti et al., 2016 [16]	Systematic review	I	90/100	Low	Groin Pain Syndrome Italian Consensus Conference on terminology, clinical evaluation, and imaging assessment in groin pain in athletes
Bisciotti et al., 2021 [19]	Systematic review	I	89/100	Low	The conservative treatment of longstanding adductor-related groin pain syndrome
Henriksen, 2016 [23]	Systematic review	I	78/100	Low	The systemic and local collagen turnover in hernia patients
Bracale et al., 2023 [25]	Systematic review	I	90/100	Low	The role of matrix metalloproteinases in the pathogenesis of inguinal hernias
Munegato et al., 2015 [28]	Systematic review	I	65/100	Moderate	The relationship between sports hernia and femoroacetabular impingement in athletes
Sheen et al., 2014 [29]	Systematic review	I	81/100	Low	The British Hernia Society’s 2014 position statement concerning the treatment of the sportsman’s groin’ based on the Manchester Consensus Conference
Swan and Wolcott, 2007 [42]	Systematic review	I	75/100	Low	Athletic hernia clinical description
Serafim et al., 2022 [46]	Systematic review	I	74/100	Moderate	The return to sport after conservative versus surgical treatment for pubalgia in athletes
Revzin et al., 2016 [51]	Systematic review	I	83/100	Low	The US examination of the inguinal canal and its correlation with CT and MR imaging
Hernia Surge Group, 2018 [52]	Systematic review	I	90/100	Low	International guidelines for groin hernia management
Caudill et al., 2008 [53]	Systematic review	I	63/100	Moderate	Clinical aspect of sport hernia
Ng et al., 2008 [54]	Systematic review	I	73/100	Moderate	The role of herniography in hernia assessment
Bisciotti et al., 2024 [55]	Systematic review	I	91/100	Low	The anatomical factors in inguinal-pubic-adductor area that may contribute to gender difference in susceptibility to groin pain syndrome
Castle et al., 2021 [45]	Retrospective case–control study	III	76/100	Low	The return to play rate following surgical management of athletic pubalgia in the national basketball population
Meyers et al., 2008 [81]	Retrospective case–control study	III	71/100	Moderate	Clinical experience concerning sport hernia
Zoga et al., 2008 [82]	Retrospective case–control study	III	70/100	Moderate	MRI findings concerning athletic pubalgia and sport hernia
Vasileff et al., 2017 [83]	Case–control study	III	86/100	Low	The role of dynamic ultrasound in sport hernia assessment
Fournier and Richon, 1993 [2]	Case series	IV	71/100	Moderate	Description of 25 patients surgically treated for sport hernia with the Nesovic technique
Van Meirhaeghe et al., 2019 [3]	Case series	IV	81/100	Low	The results of 10-year of Nesovic surgical procedure combined with adductor release for groin pain in 33 competitive athletes
Orchard et al., 1998 [11]	Case series	IV	72/100	Moderate	The role of ultrasound in inguinal canal posterior wall deficiency
Malycha and Lovell, 1992 [14]	Case series	IV	79/100	Low	Inguinal surgery in athletes with chronic groin pain
Hackney, 1993 [15]	Case series	IV	71/100	Moderate	The sports hernia: as a cause of chronic groin pain
Spangen et al., 1988 [18]	Case series	IV	78/100	Low	Non palpable inguinal hernia in the female population
Bisciotti et al., 2022 [20]	Case series	IV	90/100	Low	Multidisciplinary assessment of long-standing groin pain syndrome in athletic women in keeping with the Italian Consensus Agreement
Jorgenson et al., 2015 [26]	Case series	IV	90/100	Low	Genomic association for susceptibility loci underlying inguinal hernia.
Bisciotti et al., 2021 [33]	Case series	IV	89/100	Low	Multidisciplinary assessment of 320 athletes with long-standing groin pain syndrome in keeping with the Italian consensus agreement
Palumbo et al., 2022 [66]	Case series	IV	74/100	Moderate	Open surgery for sports
Robinson et al., 2006 [67]	Case series	IV	80/100	Low	Ultrasound examination for inguinal end femoral hernia
Ioffe et al., 2020 [68]	Case series	IV	75/100	Low	Magnetic resonance and ultrasound examination for sport hernia assessment in a football player population
van Wessem et al., 2003 [69]	Case series	IV	81/100	Low	The etiology of indirect inguinal hernias
van Veen et al., 2007 [70]	Case series	IV	70/100	Moderate	Patent processus vaginalis in the adult population as a risk factor for the indirect inguinal hernia
Wright et al., 2017 [71]	Case series	IV	77/100	Low	The neuropathy in primary inguinal hernia.
Meyers et al., 2000 [72]	Case series	IV	73/100	Moderate	Management of severe lower abdominal or inguinal pain in high-performance athletes.
Brennan et al., 2005 [73]	Case series	IV	70/100	Moderate	MRI assessment of secondary cleft sign as a marker of injury in athletes with groin pain syndrome
Falvey et al., 2016 [74]	Case series	IV	77/100	Low	Prospective anatomical diagnosis of 382 patients affected by athletic groin pain
Garner et al., 2006 [75]	Case series	IV	73/100	Moderate	The herniography examination in inguinal hernia assessment
Koch et al., 2005 [21]	Prospective study	IV	85/100	Low	Clinical evaluation of 6895 groin hernia repairs in women
Isik et al., 2017 [24]	Prospective study	IV	89/100	Low	The metalloproteinases inhibitors in patients with inguinal hernia
Dojčinović et al., 2012 [38]	Prospective study	IV	84/100	Low	Surgical treatment of chronic groin pain in athletes
Paajanen et al., 2011 [41]	Prospective study	IV	90/100	Low	Laparoscopic surgery for chronic groin pain in athletes compared to non-operative treatment
Robinson et al., 2015 [76]	Prospective study	IV	72/100	Moderate	MRI and ultrasound of the anterior pelvis and their correlation with clinical findings in a young football players population
Preskitt, 2011 [27]	Retrospective case series	IV	65/100	Moderate	The experience of Baylor University Medical Center of Dallas concerning sport hernia
Jain et al., 2010 [32]	Retrospective case series	IV	80/100	Low	Sport hernia in athletic population and its clinical presentation
Steele et al., 2004 [39]	Retrospective case series	IV	65/100	Moderate	Surgery for posterior inguinal wall weakness in athletes
Kajetanek et al., 2018 [44]	Retrospective case series	IV	67/100	Moderate	Return to play after targeted surgery for sport hernia
Kim B et al., 2015 [77]	Retrospective case series	IV	80/100	Low	Ultrasound examination in sport hernia
Garvey and Hazard, 2014 [78]	Retrospective case series	IV	85/100	Low	Sports hernia or groin disruption injury? Chronic athletic groin pain: a retrospective study of 100 patients with long-term follow-up
Lee et al., 2017 [7]	Clinical review	IV	82/100	Low	Magnetic resonance and ultrasound imaging in groin pain syndrome
Kavanagh et al., 2006 [12]	Clinical review	IV	75/100	Low	Magnetic resonance imaging in athletes affected by groin pain syndrome
Koulouris, 2008 [13]	Clinical review	IV	90/100	Low	Anatomic approach of imaging in groin pain in an elite athletic population
Elattar et al., 2016 [22]	Clinical review	IV	64/10	Moderate	Return to play after groin pain syndrome in the athletic population
Bisciotti et al., 2017 [67]	Observational cross-sectional study	IV	85/100	Low	The relationship between Cam morphology and inguinal pathologies
Bisciotti et al., 2018 [80]	Observational cross-sectional study	IV	86/90	lOW	Potential magnetic resonance imaging findings associated with inguinal hernia and inguinal canal posterior wall weakness in athletes
Bisciotti et al., 2024 [84]	Case report	IV	80/100	Low	The use of botulinum toxin in pre-pubic aponeurotic complex injuries
Nesovic, 1987 [1]	Narrative review	V	Not applicable	Not applicable	Treatment of groin pain syndrome I athletes
Zuckerbraun et al., 2020 [4]	Narrative review	V	Not applicable	Not applicable	Clinical assessment of sport hernias
Brown Aet al., 2013 [17]	Narrative review	V	Not applicable	Not applicable	Clinical concept concerning sport hernias
Balconi, 2011 [30]	Narrative review	V	Not applicable	Not applicable	Ultrasound examination in groin pain syndrome
Patel and Wright, 2021 [31]	Narrative review	V	Not applicable	Not applicable	Clinical controversies in inguinal hernia clinical assessment
Bisciotti et al., 2022 [34]	Narrative review	V	Not applicable	Not applicable	The prepubic aponeurotic complex injuries
Thorborg, 2023 [35]	Narrative review	V	Not applicable	Not applicable	Clinical concepts for adductor strains and long-standing adductor-related groin pain
Via et al., 2018 [36]	Narrative review	V	Not applicable	Not applicable	Management of osteitis pubis in athletes
Anderson, 2016 [37]	Narrative review	V	Not applicable	Not applicable	Diagnosis and treatment of iliopsoas: pathology
Minnich et al., 2011 [40]	Narrative review	V	Not applicable	Not applicable	The minimal surgical repair technique for sport hernias
Choi et al., 2016 [43]	Narrative review	V	Not applicable	Not applicable	Return to play after sports hernia surgery
Bisciotti et al., 2024 [47]	Narrative review	V	Not applicable	Not applicable	The role of magnetic resonance imaging in groin pain syndrome in athletes
Bou Antoun et al., 2018 [56]	Narrative review	V	Not applicable	Not applicable	The role of imaging in inguinal-related groin pain in athletes
Gamborg et al., 2019 [57]	Narrative review	V	Not applicable	Not applicable	Anatomical and clinical differences between inguinal hernia and sport hernia
Dimitrakopoulou and, Schilders, 2016 [58]	Narrative review	V	Not applicable	Not applicable	Anatomical and clinical definition of sport hernia
Omar et al., 2008 [59]	Narrative review	V	Not applicable	Not applicable	MRI assessment of athletes affected by athletic pubalgia and sport hernia
Shortt et al. 2008 [60]	Narrative review	V	Not applicable	Not applicable	Anatomy, pathology, and MRI assessment in subjects affected by sport hernia
Rabe and Gretchen, 2010 [61]	Narrative review	V	Not applicable	Not applicable	Treatment and prevention of athletic pubalgia
Mullens et al., 2012 [62]	Narrative review	V	Not applicable	Not applicable	MRI technique in athletic pubalgia and sport hernia
Palisch et al., 2013 [63]	Narrative review	V	Not applicable	Not applicable	Clinical and therapeutic correlations between imaging and core injuries
Moeller, 2007 [64]	Narrative review	V	Not applicable	Not applicable	Clinical concept concerning sport hernia
Chopra and Robinson, 2016 [65]	Narrative review	V	Not applicable	Not applicable	The role of imaging in groin pain syndrome in athletic population

## 3. Results

### 3.1. Definition of the Term Sport Hernia

It is important to underline that the term SH is incorrect from an anatomical point of view since no real hernia is present in this clinical situation [4]. Indeed, in a clinical situation of SH, the posterior wall of the inguinal canal does not lose its anatomical continuity as it does in a direct inguinal hernia [56,66]. Furthermore, the term SH is incorrect, as this condition may also occur in subjects used to heavy work activities but who do not necessarily practice sport [57]. From a strictly anatomical point of view the term “SH” describes a “weakness of the posterior wall of the inguinal canal” [79]. From an anatomical point of view, this last definition is more in line with the “Groin Pain Syndrome Italian Consensus Conference on terminology, clinical evaluation and imaging assessment in groin pain in athlete” [16]. Therefore, for correctness of anatomical nomenclature, the term SH should be replaced with that of ‘weakness of the inguinal canal posterior wall’ [5,16]. However, for the sake of simplicity, these two terms (i.e., SH and weakness of the inguinal canal posterior wall) will be considered as synonyms in the following text.

### 3.2. Dynamic Ultrasonography Examination

Dynamic ultrasonography examination (USD) represents the gold standard for SH investigation [5,11,16,30,51,58,67,68,76,77].

**Figure 2:**
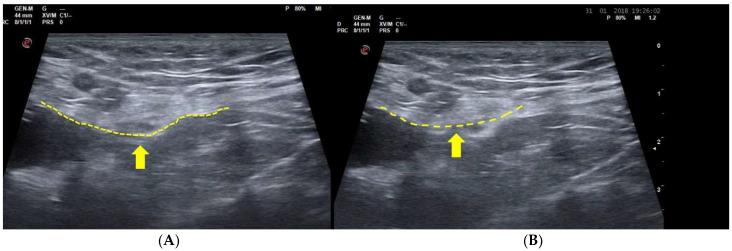
USD examination of the posterior wall of the inguinal canal. (**A**) At rest, the posterior wall shows a slight concavity (arrow); and (**B**) in physiological conditions, during the Valsalva maneuver, the same conformation is maintained (arrow).

During USD examination, the posterior wall of the inguinal canal at rest and in physiological conditions shows a slight downward concavity [5,16,19]. During the Valsalva maneuver following the increase in the abdominal pressure in physiological conditions, this same conformation is maintained (Figure 2) [5,16,19,58,68]. In other words, the posterior wall of the inguinal canal does not prolapse under an increase in intra-abdominal pressure.

**Figure 3:**
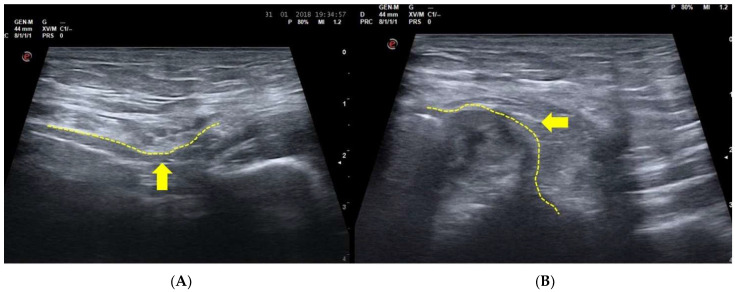
(**A**) USD examination of the posterior wall of the inguinal canal at rest (arrow); and (**B**) during the Valsalva maneuver. During the Valsalva maneuver, the physiological convexity of the posterior wall is overturned, and bulging is evident (arrow). This is a classic image of bulging indicating a posterior wall weakness without the presence of a true inguinal hernia.

**Figure 4:**
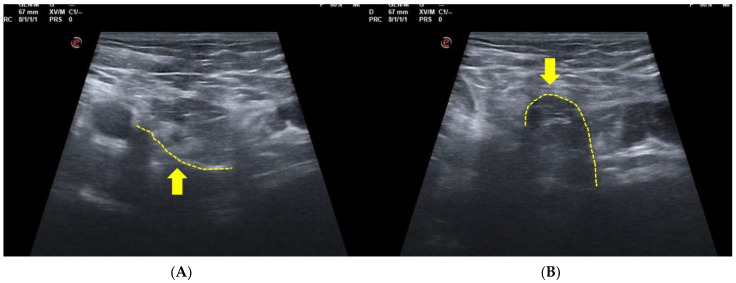
(**A**) USD examination of the posterior wall of the inguinal canal at rest; (arrow) and (**B**) during the Valsalva maneuver. During the Valsalva maneuver the seeping of preperitoneal fat which occupies the inguinal canal is evident (arrow). The image clearly indicates a direct hernia (in agreement with the European Hernia Society classification) [29].

On the contrary, in the case of posterior wall weakness of the inguinal canal, USD examination reveals that the physiological concavity of the inguinal canal posterior wall is overturned, and bulging (namely the SH) is evident during the Valsalva maneuver. In short, the concavity becomes a convexity which compresses the contents of the inguinal canal [5,11,16,30,33,58,68] (Figure 3). This is a classic image of bulging indicating a weakness of posterior wall without the presence of a true inguinal hernia [5,16,19,58,68]. On the contrary, in agreement with the European Hernia Society classification [29] in the case of a direct hernia, under USD, the posterior wall of the inguinal canal (i.e., the fascia transversalis) shows a solution of continuity (i.e., a hernia breach) from which preperitoneal fat and/or viscera originally from the inguinal canal seep during the Valsalva maneuver (Figure 4). Furthermore, it is important to remember that direct hernias occur medially to the inferior epigastric vessels [29].

In further agreement with the European Hernia Society classification [29], in the case of an external oblique (or indirect) hernia, preperitoneal fat and/or viscera seep through the internal inguinal ring and end up occupying the inguinal canal. An external oblique hernia, unlike a direct hernia, occurs laterally with respect to the inferior epigastric vessels [29].

**Figure 5:**
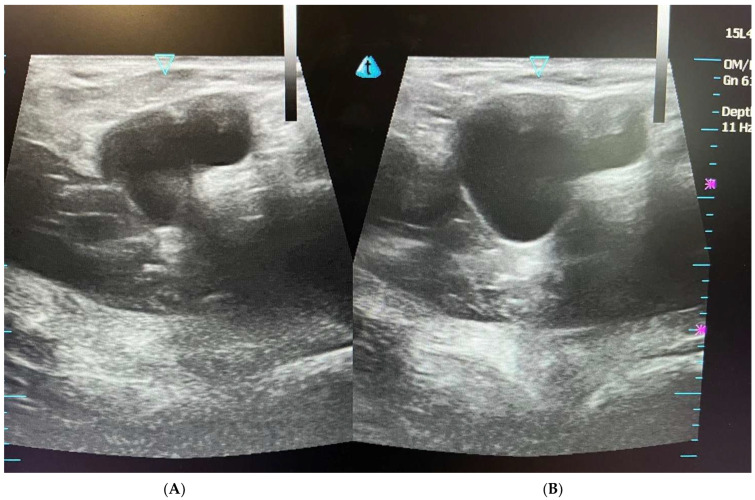
The correctness of the Valsalva maneuver must be preventively controlled by US examination of the visual distension of the femoral and iliac veins. In box (**A**), the femoral and iliac veins are visible in a resting situation. In box (**B**), the dilation of the veins during the Valsalva maneuver is visible. A satisfactory dilation of the femoral and iliac veins is indicative of a valid Valsalva maneuver.

It is important to remember that the correctness of the Valsalva maneuver must be preventively controlled by US examination of the visual distension of the femoral and iliac veins (Figure 5) [83]. During the repetition of multiple Valsalva maneuvers, the eventual movements of the tissues, medially or laterally compared to the inferior epigastric vessels, can provide the radiologist with the elements of judgment for the echographic diagnosis of a direct hernia, an external oblique hernia, and a SH. Therefore, an incorrect and/or inadequately controlled Valsalva maneuver may lead the radiologist to an erroneous diagnosis and typically to a false negative [83].

Therefore, a SH presents itself as a direct hernia, medial to the inferior epigastric vessels and at the height of the transversalis fascia. The only difference is the anatomical integrity of the transversalis fascia, which in SH presents a bulging only during the Valsalva maneuver and not a hernial breach with leakage of pre-peritoneal content or viscera as in the case of a direct hernia [5,16,19,58,68].

For these reasons, SH should be considered as a situation of incipient direct inguinal hernia (i.e., an early stage of direct hernia or a direct hernia “in fieri”) [5,14,15,16].

Therefore, the concept of “weakness of the inguinal canal posterior wall” is not only different from that of “inguinal hernia” but it is also different from that of “occult inguinal hernia” (or hidden hernia). Indeed, in agreement with the definition of the Hernia Surge Group [52] an “occult inguinal hernia” is “an asymptomatic hernia not detectable by physical examination” and for this reason, an “occult hernia” is detected only by US dynamic examination or by the examination of the contralateral asymptomatic part during laparoscopic hernia surgery [52]. Unfortunately, to date, despite this clear definition by the Hernia Surge Group [52], there is still a lack of uniformity in the definition of ‘‘occult hernia’’ in the literature. Indeed, some authors define “occult hernia” as an “actual protrusion of normally intraabdominal contents” or a “beginning hernia”, or “an incipient hernia” or a “patent processus vaginalis without herniation” [69,70]. Obviously, this lack of consensus in the definition generates a serious conceptual confusion. Therefore, it is important to have a clear and shared radiological definition during the USD assessment of the weakness of the inguinal canal posterior wall. To the best of our knowledge, the more correct definition could be as follows:

The diagnosis of posterior inguinal wall weakness can be formulated when in the USD assessment associated with a Valsalva maneuver, a clear overturning of the physiological convexity of the transversalis fascia can be observed without interruption of its anatomical continuity (i.e., a bulging without the presence of a hernial sac).

**Figure 6:**
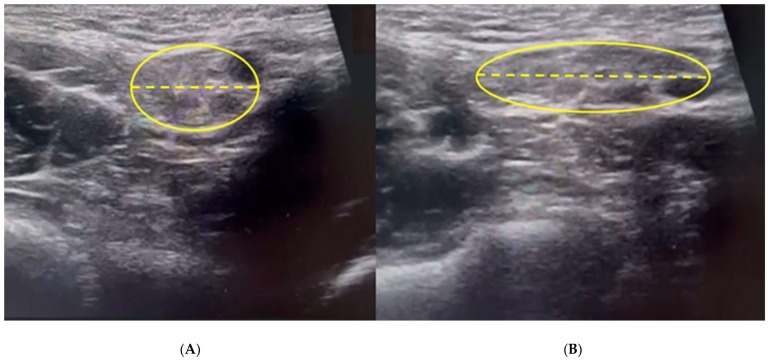
In box (**A**), USD imaging showing the external inguinal ring at rest (yellow ellipse). The image shows a dilated external ring in basal conditions with a diameter of 14 mm (dashed yellow line). In box (**B**), the USD imaging shows, during the Valsalva maneuver, a further dilatation of the external inguinal ring (yellow ellipse) whose diameter reaches 25 mm (dashed yellow line).

Furthermore, it is important to note that posterior inguinal wall weakness displays direct and indirect radiological signs upon USD examination [5,56,71]; the obvious, direct radiological evidence is the presence of a bulging during US dynamic examination performed during the Valsalva maneuver [5,56,71], whereas the indirect radiological sign is the presence of a ballooning caused by an abnormal dilatation of the external inguinal ring both in basal conditions and during the Valsalva maneuver [5,56,71]. To be precise, this ballooning is an abnormal dilatation (>1 cm) of the external inguinal ring at rest, which increases during the Valsalva maneuver (Figure 6) and is an indirect sign of both external oblique aponeurosis micro-tears and ilioinguinal nerve neuropathy [71]. This ballooning, which indicates anterior wall deficiency, arises as a consequence of degeneration and tear of the external oblique muscle and aponeurosis and results in a dehiscence between the inguinal ligament leading to dilatation of the superficial inguinal ring [56]. However, it is necessary to specify that the ballooning sign is also present in the presence of a direct or indirect hernia [5,56,71].

**Figure 7:**
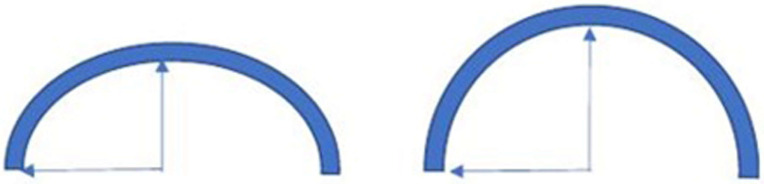
Upon USD examination, a circular arc with height ≤ radius (h ≤ r) is suggestive of a weakness of the inguinal canal posterior wall.

**Figure 8:**
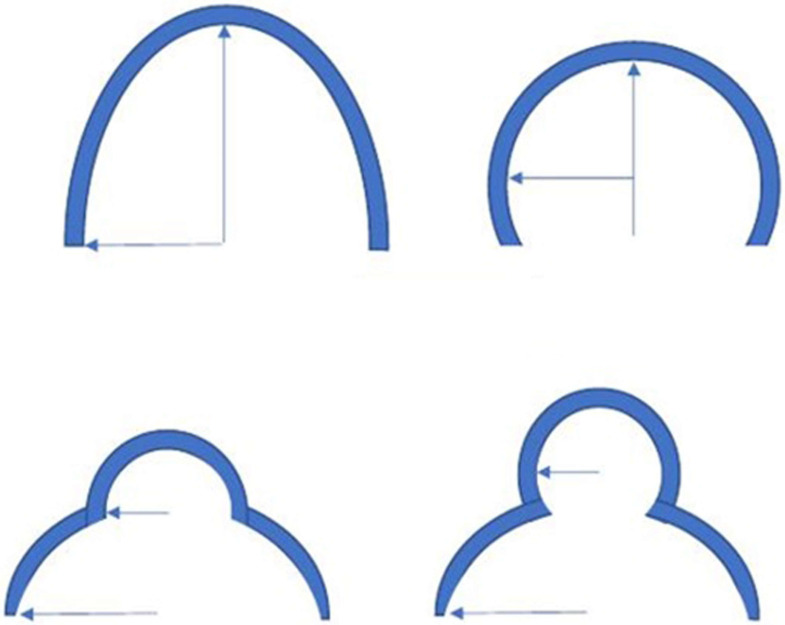
On the contrary, if the USD imaging shows that the arc in which h > r or the bulging is interrupted by a line with a smaller radius of curvature, the imaging is suggestive of a true hernia.

A correlation between anatomy and USD imaging may be found to discern the weakness of the inguinal canal posterior wall and a true hernia [5]. A proposed distinction could be the shape of bulging. If, during the Valsalva maneuver, an arc of a circle is observed with height ≤ radius (h ≤ r), the image is suggestive of inguinal canal posterior wall deficiency [5] (Figure 7). On the contrary, if during the Valsalva maneuver the arc observed is of the type h > r, or the bulging is interrupted by a line with a smaller radius of curvature, the image is suggestive of a true hernia [5] (Figure 8).

### 3.3. Magnetic Resonance Imaging

Although the use of magnetic resonance imaging examination (MRI) is recommended as a second level examination [52], not all authors consider this advisable [5,7,16,78]. Indeed, this examination is often ineffective and does not lead to a diagnosis because of the difficulty many patients experience in performing the Valsalva maneuver correctly, especially if there is no direct control of the maneuver itself by the technical operator. On the contrary, the correct real-time execution of the Valsalva maneuver during USD makes for the gold standard in SH investigation [5,11,16,30,51,58,67,68,76,77]. In this context, it is important to remember that an US static examination, which does not involve a Valsalva maneuver, fails to spot the majority of inguinal hernias and/or inguinal posterior wall weakness [53,83].

Nonetheless, MRI is the diagnostic test of choice concerning the bone, muscle, and ligament structures of the symphysis for lesions of the ilio-inguinal ligament and the conjoint tendon [56]. Indeed, the anatomical value of MRI examination in inguinal pathologies is not questioned but its functional validity (i.e., during some functional maneuvers such as the Valsalva maneuver) may be challenged [56]. Indeed, in the specific case of the Valsalva maneuver in the investigation of hernias or of the posterior inguinal wall deficiency, MRI can give rise to an unacceptable number of false negatives [56].

**Figure 9:**
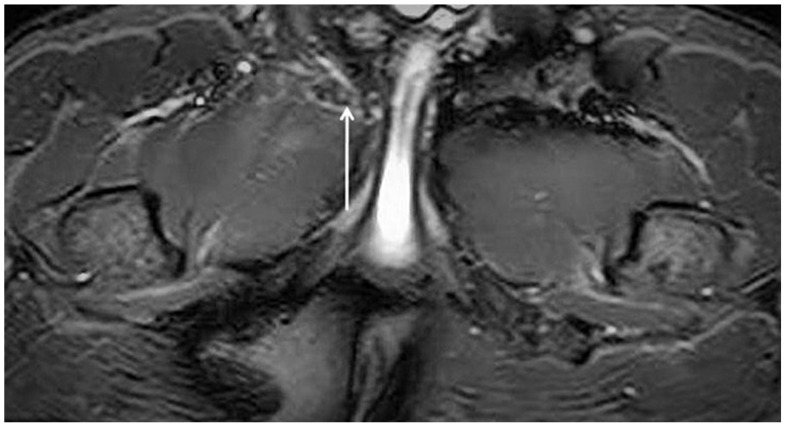
Axial oblique PD FS MRI showing a right adductor longus tendinopathy (arrow).

**Figure 10:**
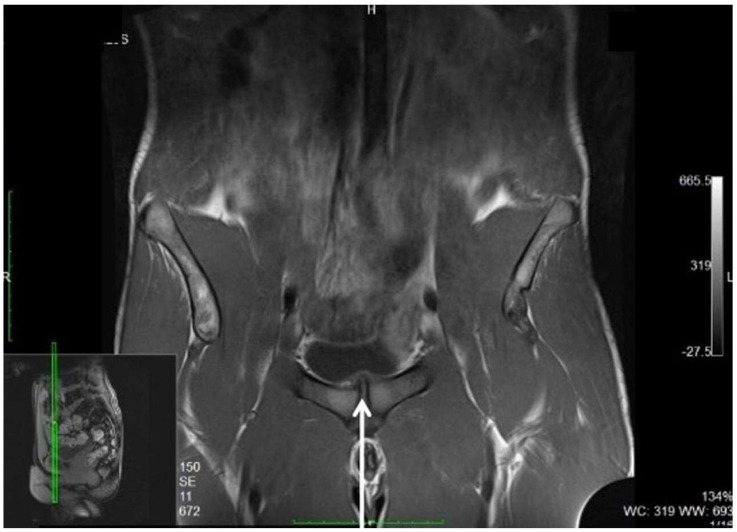
Coronal T1-MRI showing a symphyseal central disc protrusion (arrow).

**Figure 11:**
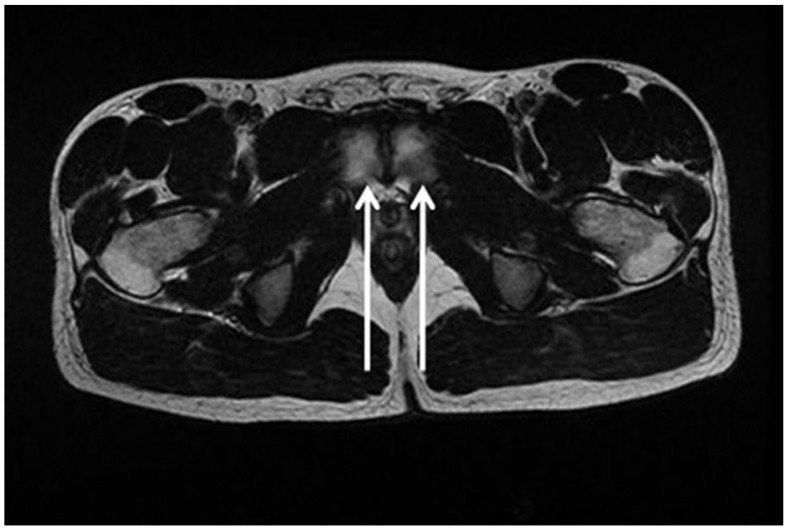
Axial oblique T2 MRI showing bone marrow oedema extended across the surface, in the antero-posterior direction of both the pubic branches (arrows).

Despite this, MRI examination of the pelvis provides important indirect signs of the presence of inguinal posterior wall weakness. Specifically, these are the following:

(i) Adductor longus tendinopathy detected upon MRI examination (Figure 9) is the most important radiological sign correlated with inguinal posterior wall weakness (OR 3.83; 1.27 to 11.54; 95% CI) [80].
(ii) Symphyseal central disc protrusion (Figure 10) represents the second most important radiological sign associated with the presence of inguinal posterior wall weakness (OR 3.77; 1.19 to11.92; 95% CI) [80].
(iii) Finally, the presence of bone marrow oedema of the pubic branches (Figure 11) is the third most important sign in the MR investigation strongly associated with the presence of inguinal posterior wall weakness (OR 3.68; 0.74 to 18.23; 95% CI) [80].

It is interesting to note that most of the MRI-based studies regarding the onset of GPS focus on other clinical situations and not on SH or inguinal hernias. Meyers et al. [72,81] relied on MRI in their studies to overcome the pitfalls often encountered in GPS diagnosis, whilst Brennan et al. [73] underlined the importance of the secondary cleft sign as a marker of GPS in afflicted athletes. Omar et al. [59] recognized the importance of performing USD and MRI during the Valsalva maneuver in order to visualize inguinal hernias and SH better. Unfortunately, once again, we are faced with the incumbrance of not being able to check the correctness of the Valsalva maneuver in real-time during MRI examination [56]. Zoga et al. [82] in their study focused on the discordance between MRI and the surgical findings, and they highlighted the rectus abdominis insertional injury as frequently giving false negative imaging. Other authors [60,61], in emphasizing the importance of MRI in GPS diagnosis, commit the serious conceptual error of considering ‘SH’ as a broad term for a whole spectrum of pubic symphysis pathologies that cause GPS. Mullens et al. [62] commit another conceptual mistake by erroneously identifying the SH as a distal rectus abdominis detachment/tearing from the pubic ramus accompanied by a concomitant partial or complete tear of the adductor longus origin. Finally, Palisch et al. [63] evoke the importance of MRI in what they generically define as “core injuries” namely numerous musculoskeletal and visceral injuries including SH.

These observations, in addition to reminding us of the conceptual confusion associated with the term ‘SH’, call for three important points to be outlined:

(i) The poor validity of MRI in the diagnosis of inguinal hernias and SH due to problems inherent to the Valsalva maneuver [56].
(ii) The importance of MRI investigation for the study of other clinical situations that may be the cause of GPS concerning bone, muscle, and ligament structures of the symphysis, and lesions of the ilio-inguinal ligament and the conjoint tendon [56,73,81].
(iii) The different diagnostic approaches to GPS shown by different authors can be explained, at least partially, both by the fact that the diagnostic approach was performed on different sporting cohorts and/or graphical populations, and by the notable difference in the interpretation of the clinical examinations [74].

### 3.4. Herniography

Herniography is a diagnostic invasive examination that involves injecting a contrast medium into the peritoneal cavity in order to visualize the inguinal region [72]. Herniography was widely used in the past for diagnosing inguinal hernias due to its high diagnostic sensitivity and specificity [54]. However, its use today, as a first-line diagnostic technique, is not recommended because of its invasiveness [10,75].

### 3.5. Other Imaging Techniques

Conventional radiology CT scan imaging and bone scintigraphy are imaging methods that have a high sensitivity for bone pathologies but no application in the study of inguinal pathologies such as hernias or weaknesses of the posterior wall of the inguinal canal [10,16].

### 3.6. What Does the Radiologist Look for to Define SH and GPS?

From the analysis of current literature, two fundamental data emerge. The first is represented by the fact that USD represents the gold standard examination for the study of inguinal hernias and SH. This is justified by the fact that the radiologist can check, in real time, the correctness of the Valsalva maneuver, which may be requested multiple times if necessary, and this represents the foundation for the diagnosis of both inguinal hernias and SH [5,11,16,30,33,58,68,83]. The second point is based on the fact that GPS can be induced by numerous clinical situations, many of which are not investigable with US or USD techniques [5,16]. For this reason, the radiologist must complement the US and USD examinations with MRI examinations in order to reach a definitive diagnosis. Therefore, in the context of GPS diagnosis, US, USD, and MRI represent complementary and irreplaceable examinations for a complete diagnosis [5,16,56,72,81,83].

## 4. Discussion

Sports patients complaining of long-standing GPS [16] (i.e., chronic GPS) show a high prevalence of inguinal posterior wall weakness in comparison to an asymptomatic control population [19,20,83].

The mechanism that generates pain in SH is exactly identical to that implicated in the direct or external oblique inguinal hernias. In both these latter cases, the pre-peritoneal adipose tissue or, in case of SH, the bulging cause a “mass effect” that compresses the nerve endings of the iliohypogastric-, ilioinguinal-, and the genitofemoral-nerves that pass through the inguinal canal triggering the onset of peripheral neuropathy [37,40]. However, it is difficult to diagnose an inguinal posterior wall weakness by clinical examination alone [11,64,83]. Indeed, the clinical presentation of SH is that of an impalpable inguinal bulging, which can be detected only with USD examination [78]. This clinical situation is the exact opposite of that of a “classic” inguinal hernia (direct or external oblique), where there is a clinically palpable defect, which increases with an increase in abdominal pressure [78]. For this reason, the diagnosis of SH is primarily radiological [78] and USD examination is an irreplaceable means of diagnostic investigation [53]. In this regard, it is important to remember that a US static examination that does not involve the Valsalva maneuver may not pick up on the majority of inguinal hernias and/or inguinal posterior wall weaknesses [53,83]. For this reason, it is precautionary and extremely important to check the correctness of the Valsalva maneuver via an ultrasound examination, which can verify a suitable dilation of the femoral and iliac veins [83].

It is important to remember that the presence of an abnormal dilatation of the external inguinal ring represents an indirect sign of a possible weakness in the posterior wall of the inguinal canal [5,56,71]. Furthermore, it is important to underline that the ability to differentiate between a weakness of the posterior wall of the inguinal canal and an inguinal hernia by means of USD imaging is a source of valuable information important for designing an appropriate surgical plan. However, an important problem associated with USD diagnosis of SH is that a correct diagnosis can only be achieved with the intervention of an appropriately trained ultrasound operator [5,16,78].

USD examination cannot be validly replaced by MRI examination in the diagnosis of SH [5,7,16,78]. Indeed, the impossibility of checking the correctness of Valsalva maneuvers in real time during the MRI examination makes the number of false negatives unacceptable [5,56,83]. However, since SH typically coexists with multiple pathologies such as adductor longus tendinopathy, marrow oedema of the pubic branches [80], pubic osteopathy [55,78,84] and cam-morphology [79], MRI examination remains an excellent means of investigation for correctly completing diagnoses [47,65]. Furthermore, MRI may also provide important secondary clues that lead the radiologist to suspect the presence of an inguinal pathology which can then be verified via USD examination [80].

Finally, it should be remembered that the radiographic presence of SH detected via USD examination in asymptomatic subjects where GPS is not implicated, accounts for 16 and 20% of subjects [11,78]. For this reason, the radiological diagnosis of SH must necessarily be correlated with a concordant clinical presentation [29].

## 5. Conclusions

SH is best defined as a weakness of the posterior wall of the inguinal canal which, in response to a Valsalva maneuver, loses its posterior convexity and forms a bulging that compresses the nerves passing along the inguinal canal thus causing the onset of peripheral neuropathy. From a strictly anatomical point of view, SH represents a direct inguinal hernia “in fieri”. The presence of an excessive dilation of the external inguinal ring is an indirect sign of the possible presence of posterior inguinal canal wall weakness. Finally, it is important to remember that US, USD, and MRI examinations complement each other and are capable of providing different radiological information essential for a sure diagnosis of GPS.

## Data Availability

In a systematic review there is no research data to share.
